# Complete mitochondrial genome of the female-wingless bagworm moth, *Eumeta variegata* Snellen, 1879 (Lepidoptera: Psychidae)

**DOI:** 10.1080/23802359.2018.1511851

**Published:** 2018-10-08

**Authors:** Jun Seong Jeong, Min Jee Kim, Sung Soo Kim, Iksoo Kim

**Affiliations:** a Department of Applied Biology, College of Agriculture & Life Sciences, Chonnam National University, Gwangju, Republic of Korea;; b Research Institute for East Asian Environment and Biology, Seoul, Republic of Korea

**Keywords:** Mitochondrial genome, Psychidae, *Eumeta variegata*, phylogeny

## Abstract

The complete mitochondrial genome of the female-wingless bagworm moth, *Eumeta variegata* Snellen, 1879 (Lepidoptera: Psychidae), is 15,660 base pairs (bp) and contains a typical set of genes (13 protein-coding genes [PCGs], 2 rRNA genes, and 22 tRNA genes) and one non-coding region, with an arrangement identical to that observed in most lepidopteran genomes. Twelve PCGs contained the typical ATN start codon, whereas *COI* had the atypical CGA codon, which is frequently detected in the start region of the lepidopteran *COI*. The A + T-rich region was unusually short with only 94 bp. A recent report of the same species originating from Japan revealed a lack of *trnE* and *trnF* and a 1,118 bp long A + T-rich region. Phylogenetic analyses with concatenated sequences of the 13 PCGs and two rRNA genes using the Bayesian inference method placed *E. variegata* in Psychidae, as a sister to a within-familial species, *Mahasena colona*, with the highest nodal support (Bayesian posterior probability = 1).

The female-wingless bagworm moth, *Eumeta variegata* Snellen, 1879 (Lepidoptera: Psychidae), is distributed in Australia and Asia, including in Korea (Turner 1947; Robinson et al. 1994; Sobczyk 2011). In Korea, the species was named as *E. japonica* Heylaerts, 1884, but it has recently been synonymized as *E. variegata* (Roh et al. 2016). The larvae of this species make case for larval and pupal stages (Zhang 1997; Gries et al. 2006). Although the adult males have wings, female wings are reduced or missing, and thus the females and larvae have a similar appearance (Niitsu et al. 2008).

An adult male *E. variegata* was collected from Gageodo, Jeollanamdo Province (34°04′38″N, 125°06′10″E), South Korea in 2013. This voucher specimen was deposited at the Chonnam National University, Gwangju, Korea, under accession no. CNU5692. Using DNA extracted from the hind legs, three long overlapping fragments (LFs; *COI*-*ND4*, *ND5*-*lrRNA*, and *lrRNA*-*COI*) were amplified using previously described primers (Kim et al. 2012). These three LFs were used as templates to amplify 26 short fragments (Kim et al. 2012).

Phylogenetic analysis using the concatenated nucleotide sequences of 13 protein-coding genes (PCGs) and two rRNA genes was performed using a Bayesian inference (BI) method implemented in CIPRES Portal v. 3.1 (Miller et al. 2010). An optimal partitioning scheme (six partitions) and substitution model (GTR + Gamma + I) were determined using PartitionFinder 2 and the Greedy algorithm (Lanfear et al. 2012, 2014, 2016).

The complete 15,660 base pair (bp) mitochondrial genome (mitogenome) of *E. variegata* was composed of typical gene sets (two rRNAs, 22 tRNAs, and 13 PCGs) and a major non-coding A + T-rich region (GenBank accession no. MH574939). The length of the *E. variegata* A + T-rich region was the shortest at 94 bp among sequenced Tineoidea (728–1610 bp; data not shown). The gene arrangement of *E. variegata* is identical to that of the ditrysian Lepidoptera with *trnM*-*trnI*-*trnQ* between the A + T-rich region and *ND2* junction (Kim et al. 2010). Twelve PCGs contained the typical ATN start codon, whereas *COI* showed an atypical CGA codon frequently found in the start region of the lepidopteran *COI* (Kim et al. 2010). Recently, the complete mitogenome of the same species originating from Japan was reported (Arakawa et al. 2018). The overall sequence identity between the *E. variegata* described in this study and that from Arakawa et al. (2018) for PCGs and rRNAs was high, ranging from 97.81% (*srRNA*) to 99.39% (*CytB*) after removing non-overlapping sequences at the beginning and end of each gene. However, the two sequences differed in gene content; the species described by Arakawa et al. (2018) contained only 20 tRNAs, and was missing *trnE* and *trnF*. Further, the A + T-rich region of our *E. variegata* mitogenome sequence was 94 bp, whereas that of the species described by Arakawa et al. (2018) was 1118 bp, including 121 bp eight repeat sequences. Our 94 bp A + T-rich region sequence was well-aligned at the beginning and end regions with the sequence reported by Arakawa et al. (2018).

**Figure 1.**
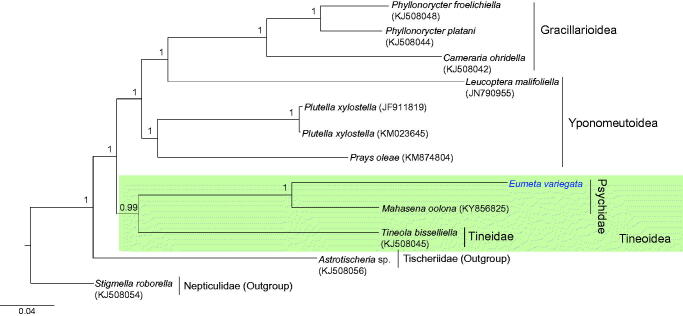
Phylogenetic tree of Ditrysia (Tineoidea, Gracillarioidea, Yponomeutoidea), including *E. variegata*. The tree was constructed using nucleotide sequences of 13 protein-coding genes and two rRNAs with the Bayesian inference (BI) method. The numbers at each node specify Bayesian posterior probabilities. Scale bar indicates the number of substitutions per site. *Astrotischeia* sp. (Nepticuloidea; Tischeriidae) and *Stigmella roborella* (Nepticuloidea; Nepticulidae) were used as outgroups. GenBank accession numbers are as follows: *Mahasena oolona*, KY856825 (Li et al. 2017); *Tineola bisselliella*, KJ508045 (Timmermans et al. 2014); *Phyllonorycter platani*, KJ508044 (Timmermans et al. 2014); *P. froelichiella*, KJ508048 (Timmermans et al. 2014); *Cameraria ohridella*, KJ508042 (Timmermans et al. 2014); *Prays oleae*, KM874804 (van Asch et al. 2014); *E. variegata*, MH574939 (This study); *Leucoptera malifoliella*, JN790955 (Wu et al. 2012); *Plutella xylostella*, JF911819 (Wei et al. 2013); *P. xylostella*, KM023645 (Dai et al. 2016); *Astrotischeria* sp., KJ508056 (Timmermans et al. 2014); and *Stigmella roborella*, KJ508054 (Timmermans et al. 2014).

Phylogenetic analysis revealed a sister relationship between *E. variegata* and the within-familial species *Mahasena colona* (Li et al. 2017) with the highest nodal support (Bayesian posterior probability = 1). Currently, mitogenome sequences are available for only three species in two families in Tineoidea, including *E. variegata*. Thus, additional mitogenome sequences from a diverse taxonomic group are required to infer the relationships among families in Tineoidea.
